# Social Determinants of Health and the Availability of Cancer Clinical Trials in the United States

**DOI:** 10.1001/jamanetworkopen.2024.10162

**Published:** 2024-05-07

**Authors:** Rishi Robert Sekar, Lindsey Allison Herrel, Kristian Donald Stensland

**Affiliations:** 1 Department of Urology, University of Michigan, Ann Arbor; 2 Institute for Healthcare Policy and Innovation, University of Michigan, Ann Arbor; 3 National Clinician Scholars Program, University of Michigan, Ann Arbor

## Abstract

This cohort study investigates whether county-level social determinants of health are associated with cancer clinical trial availability in the United States.

## Introduction

Despite dramatic growth in the number of cancer clinical trials over the past decade, disparities in enrollment persist across minoritized and historically marginalized populations, remaining a key barrier to achieving equitable cancer care.^1,2,3^ Adverse social determinants of health (SDOH; eg, economic stability, education, community infrastructure) and reduced geographic availability of trials likely interact to worsen this disparity, warranting improved understanding of this dynamic to guide expansion of trial opportunities.^4,5^ For these reasons, we evaluated the association between county-level SDOH and cancer clinical trial availability in the United States.

## Methods

This cohort study was exempt from institutional review board review in accordance with 45 CFR §46 as data used in this study were deidentified and publicly available. We followed the STROBE reporting guideline. Using data linkage from ClinicalTrials.gov, Surveillance, Epidemiology, and End Results (SEER; 2020 data), and the Centers for Disease Control and Prevention Social Vulnerability Index (SVI; 2018 data)^6^ as a county-level measure of SDOH (eMethods in Supplement 1), we performed cross-sectional and longitudinal analyses of county-level trial availability and SDOH. We included phase 2 and phase 3 interventional trials from 2007 to 2022 for the most common cancers (prostate, breast, lung, colorectal, bladder, uterine, kidney, and melanoma). Primary outcomes of interest included 2 measures of trial availability: (1) the presence of any trial within a county (dichotomous variable) and (2) the total number of trials within a county (population-adjusted for 100 000 residents ≥50 years of age; count variable), both calculated over the study period. Counties were stratified into SVI quintiles (ie, least [first quintile] to most [fifth quintile] socially vulnerable). Multivariable logistic and negative binomial regression analyses were performed to evaluate the association between SVI and trial availability, adjusting for county population, cancer incidence, and presence of Commission on Cancer (CoC) hospitals. A time plot was created to assess trends in trial availability by SVI over the study period. Data were analyzed using Stata SE version 18 (StataCorp). Two-sided *P* < .05 was considered statistically significant.

## Results

**Figure 1.**
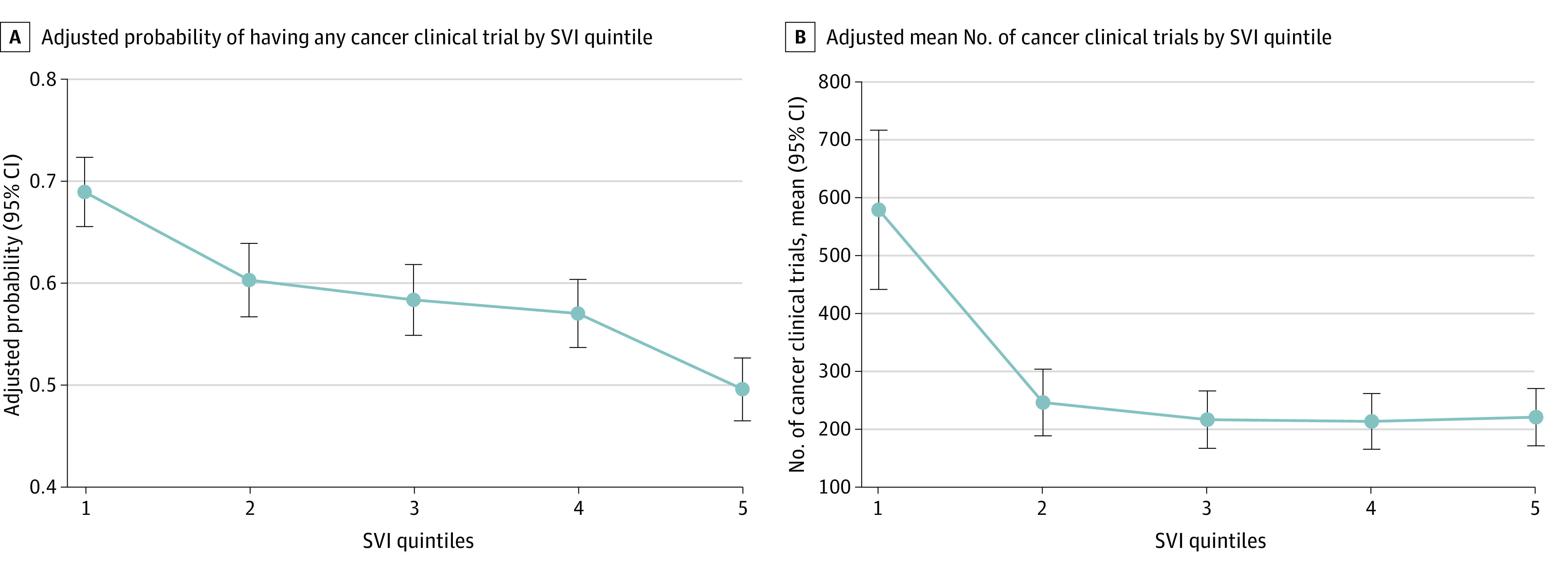
Adjusted Probability of Having Any Clinical Trial and Adjusted Mean Number of Cancer Clinical Trials by Social Vulnerability Index (SVI) Quintile The figures show the adjusted probability of having any clinical trial by SVI quintile and the adjusted mean number of cancer clinical trials by SVI quintile, adjusting for county population, cancer incidence rates, and presence of Commission on Cancer hospitals.

**Figure 2.**
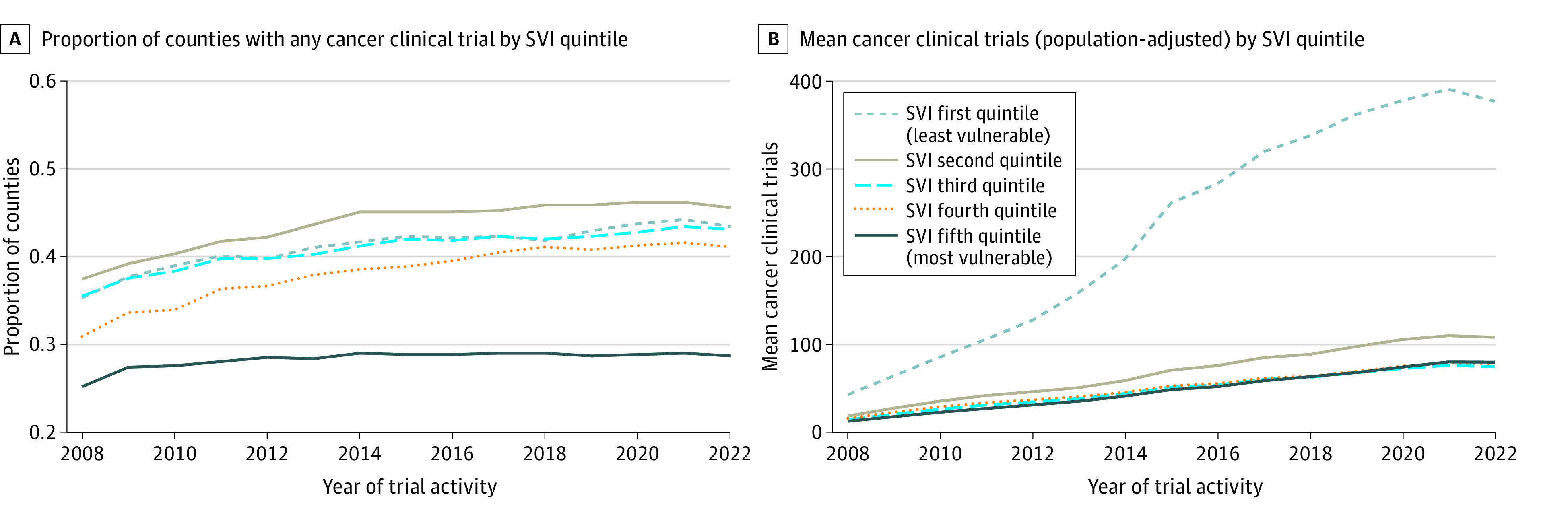
Time Plot of the Proportion of Counties With Any Cancer Clinical Trial and Mean Number of Population-Adjusted Cancer Clinical Trials by Social Vulnerability Index (SVI) Quintile The time plots (2007 to 2022) demonstrate the proportion of counties with any cancer clinical trial by SVI quintile and the mean number of population-adjusted (per 100 000 residents ≥50 years of age) cancer clinical trials by SVI quintile. Relevant rules and regulations for reporting of clinical trials in ClinicalTrials.gov: Food and Drug Administration Amendments Act (2007), Health and Human Services Final Rule (2017), and National Institutes of Health (NIH) Policy on the Dissemination of NIH-Funded Clinical Trial Information (2017).

Among 3142 US counties with a mean (SD) SVI of 0.50 (0.29) and a median (IQR) population of 10 163 (4448-26 107) people included in this study, 144 counties (4.6%) had a CoC hospital. Over the study period, 1834 counties (58.4%) had any trial, while the mean (SD) number of county-level population-adjusted trials was 342.1 (1486.1). On multivariable analysis of 2908 counties with available SEER cancer incidence data, the most socially vulnerable counties were less likely to have any trial (49.6% vs 70.0%; odds ratio, 0.33 [95% CI, 0.25-0.43]; *P* < .001) (Figure 1A) and had fewer population-adjusted trials (223.4 vs 579.2; incidence rate ratio, 0.39 [95% CI, 0.28-0.53]; *P* < .001) (Figure 1B) compared with the least socially vulnerable counties. A time plot demonstrated these observed associations across SVI quintiles throughout the study period, with stable differences in the proportion of counties with any trial (Figure 2A), but a relative increase in the mean number of trials for the least socially vulnerable counties compared with the rest (Figure 2B).

## Discussion

Substantial geographic disparities in cancer clinical trials availability exist throughout the United States, with the most socially vulnerable counties being far less likely to have any trial and having only a fraction of trials available, a disparity that has worsened over time. This study contributes new perspectives to the role of SDOH in disparities in clinical trial participation by exploring community-level measures of SDOH via SVI, providing a national-level analysis, and demonstrating trends over the past 15 years. Limitations of this study include not accounting for patient-level factors, patient travel beyond their county of residence, or the decision to enroll in a trial. Despite these limitations, this study emphasizes the role of SDOH in the disparate availability of cancer clinical trials and demonstrates the need to identify socially vulnerable communities for expansion of trial opportunities toward improving representation in studies and ensuring equitable cancer care.

## Notes

Supplement 1.
eMethods.

Supplement 2.
Data Sharing Statement
