# Supplementary material for: Social Determinants of Health and the Availability of Cancer Clinical Trials in the United States
Source: JAMA Netw Open. 2024 May 7;7(5):e2410162. doi: 10.1001/jamanetworkopen.2024.10162 (PMC11077395; doi:10.1001/jamanetworkopen.2024.10162)
Supplement: Supplement 1. — eMethods. [file jamanetwopen-e2410162-s001.pdf]

Supplemental Online Content

Sekar RR, Herrel LA, Stensland KD. Social determinants of health and the availability of cancer clinical trials in the United States. *JAMA Netw Open.* 2024;7(5):e2410162. doi:10.1001/jamanetworkopen.2024.10162

eMethods. Supplemental Methods

This supplemental material has been provided by the authors to give readers additional information about their work.

© 2024 Sekar RR et al. *JAMA Network Open.*

eMethods. **Supplemental Methods**

For the purposes of this study, the Centers for Disease Control and Prevention Social Vulnerability Index (SVI, 2018 data) was utilized as an area-level measure of social determinants of health. The SVI indicates the relative vulnerability of every United States census tract, which are subdivisions of counties for which the Census collects statistical data via the American Community Survey. The SVI is also provided at the county-level which we have utilized for the purposes of this study. The SVI is derived from 15 variables from American Community Survey (table below) which include themes of socioeconomic status, household composition and disability, minority status and language, and housing type and transportation. For each county, an overall SVI is calculated based on percentile rankings, with a higher SVI indicating higher vulnerability.[6]

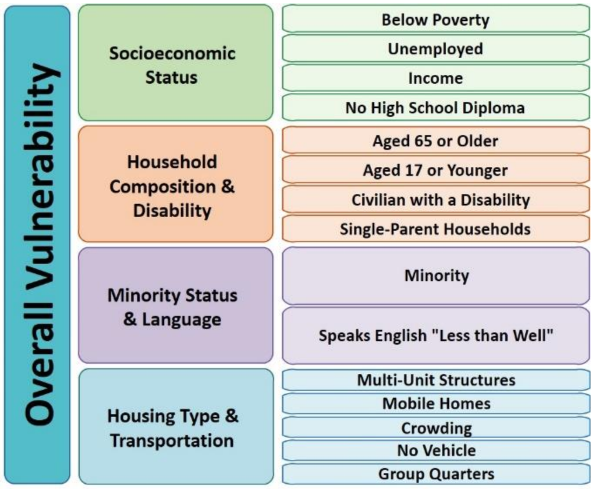

© 2024 Sekar RR et al. *JAMA Network Open.*
