# Supplementary material for: Social Determinants of Health and the Availability of Cancer Clinical Trials in the United States
Source: JAMA Netw Open. 2024 May 7;7(5):e2410162. doi: 10.1001/jamanetworkopen.2024.10162 (PMC11077395; doi:10.1001/jamanetworkopen.2024.10162)
Supplement: Supplement 2. — Data Sharing Statement [file jamanetwopen-e2410162-s002.pdf]

Data Sharing Statement

Sekar. Social Determinants of Health and the Availability of Cancer Clinical Trials in the United States. *JAMA Netw Open*. Published May 07, 2024.
doi:10.1001/jamanetworkopen.2024.10162

Data
Data available: No

Additional Information
Explanation for why data not available: Data utilized for this study is publicly available.
